# Using the Low-Income Housing Tax Credit to Fund Assisted Living: A Mixed-Methods Study

**DOI:** 10.1093/geroni/igaf122.3197

**Published:** 2025-12-31

**Authors:** Jordan Kaplan, Rebecca Smith, Taylor Craig, Paula Carder, Janiece Taylor, Gauri Gadkari, Craig Pollack, Kali Thomas

**Affiliations:** Johns Hopkins University School of Medicine, Baltimore, Maryland, United States; Johns Hopkins University, Baltimore, Maryland, United States; Johns Hopkins University, Baltimore, Maryland, United States; Portland State University, Portland, Oregon, United States; Johns Hopkins University, Baltimore, Maryland, United States; Brown University, Providence, Rhode Island, United States; Johns Hopkins University, Baltimore, Maryland, United States; Johns Hopkins University, Baltimore, Maryland, United States

## Abstract

In the United States, over 31,000 assisted living communities (ALs) serve over 950,000 Americans, providing them with personal care and assistance. At an average monthly cost of $4,500, ALs are often out of reach for middle and low-income older adults. We identified ALs serving low-income populations by focusing on the Low-Income Housing Tax Credit (LIHTC), the country’s largest funder of affordable units. We matched an inventory of ALs to HUD’s database of LIHTC developments. To identify barriers and opportunities, we conducted semi-structured interviews with 25 stakeholders in roles related to affordable AL development. We identified 197 LIHTC-funded ALs concentrated in a handful of states including Massachusetts (n = 31), Indiana (n = 26), Iowa (n = 13), Colorado (n = 12), and Maine (n = 12). Thirteen states lacked any LIHTC-ALs. LIHTC-funded ALs were more likely to be in a medically underserved areas compared to other ALs (12% vs 5.8%, p < 0.001). Themes identified from interviews include: (1) LIHTC can be paired with other funding streams to build affordable AL in the right regulatory environment; (2) sufficient Medicaid reimbursement rates are critical to a developer’s success; and (3) affordable AL development often requires both LIHTC and Medicaid funds, and the complexity of pairing the two often stymies development. In summary, we conducted a novel data match and found LIHTC has been used to fund affordable AL in 37 states. Themes identified from qualitative analysis underscore the extent to which state-level factors affect the viability of LIHTC-AL development.

